# Dual-specificity phosphatase 5 acts as an anti-inflammatory regulator by inhibiting the ERK and NF-κB signaling pathways

**DOI:** 10.1038/s41598-017-17591-9

**Published:** 2017-12-11

**Authors:** Huiyun Seo, Young-Chang Cho, Anna Ju, Sewoong Lee, Byoung Chul Park, Sung Goo Park, Jeong-Hoon Kim, Kwonseop Kim, Sayeon Cho

**Affiliations:** 1 College of Pharmacy, Chung-Ang University, Seoul, 06974 Republic of Korea; 2 Disease Target Structure Research Center, Korea Research Institute of Bioscience and Biotechnology, Daejeon, 34141 Republic of Korea; 3 Personalized Genomic Medicine Research Center, Korea Research Institute of Bioscience and Biotechnology, Daejeon, 34141 Republic of Korea; 4 College of Pharmacy and Research Institute for Drug Development, Chonnam National University, Gwang-ju, 61186 Republic of Korea

## Abstract

Although dual-specificity phosphatase 5 (DUSP5), which inactivates extracellular signal-regulated kinase (ERK), suppresses tumors in several types of cancer, its functional roles remain largely unknown. Here, we show that DUSP5 is induced during lipopolysaccharide (LPS)-mediated inflammation and inhibits nuclear factor-κB (NF-κB) activity. *DUSP5* mRNA and protein expression increased transiently in LPS-stimulated RAW 264.7 cells and then returned to basal levels. DUSP5 overexpression in RAW 264.7 cells suppressed the production of pro-inflammatory tumor necrosis factor-alpha (TNF-α) and interleukin-6 (IL-6), whereas knockdown of *DUSP5* increased their expression. Investigation of two major inflammatory signaling pathways, mitogen-activated protein kinase (MAPK) and NF-κB, using activator protein-1 (AP-1) and NF-κB reporter plasmids, respectively, showed that NF-κB transcription activity was downregulated by DUSP5 in a phosphatase activity-independent manner whereas AP-1 activity was inhibited by DUSP5 phosphatase activity towards ERK,. Further investigation showed that DUSP5 directly interacts with transforming growth factor beta-activated kinase 1 (TAK1) and inhibitor of κB (IκB) kinases (IKKs) but not with IκBα. DUSP5 binding to IKKs interfered with the association of TAK1 with IKKs, suggesting that DUSP5 might act as a competitive inhibitor of TAK1-IKKs association. Therefore, we propose that DUSP5 negatively regulates ERK and NF-κB in a phosphatase activity-dependent and -independent manner, respectively.

## Introduction

Phosphorylation of serine, threonine, or tyrosine residues in proteins is a typical post-translational modification in eukaryotes that is a critical part of signal transduction pathways involved in important cellular processes such as cell differentiation, proliferation, apoptosis, gene expression, cytoskeletal function, and immunological signaling^1^. Protein phosphorylation is regulated by the equal and balanced action of protein kinases and phosphatases in mammalian cells^2^.

Macrophages are innate immune cells activated during microbial infection and are vital mediators of innate immune responses such as phagocytosis, antigen presentation, and secretion of cytokines, chemokines, and several other factors^3^. Macrophages stimulated by lipopolysaccharide (LPS) release pro-inflammatory cytokines such as tumor necrosis factor-alpha (TNF-α), interleukin-6 (IL-6), IL-12, monocyte chemotactic protein-1, interferon-gamma, and IL-10 through complex signaling mechanisms^4^. Stimulated macrophages and dendritic cells localized to affected tissues recognize pathogen-associated molecular patterns via specific receptors, including Toll-like receptors and nucleotide-binding oligomerization domain-containing proteins^5,6^. Then, adaptor proteins, including myeloid differentiation factor 88 (MYD88) and Toll/IL-1 receptor domain-containing adapter protein inducing interferon-β (TRIF), in turn activate the mitogen-activated protein kinase (MAPK) and nuclear factor-κB (NF-κB) pathways^7^. MAPKs induce cytokine gene expression by promoting transcription factors such as activator protein-1 (AP-1), which enhance the stability of cytokine and chemokine mRNAs^8^. In addition, as a transcription factor that binds to the promoter region of many inflammatory cytokines, NF-κB acts as a key player in the regulation of inflammatory response genes^9^. NF-κB is inactivated in the cytoplasm through association with inhibitory proteins like inhibitor of κB (IκB) α/β in resting macrophages. Stimulation of macrophages with LPS activates an IκB kinase (IKK) complex that contains three subunits designated IKKα, IKKβ, and IKKγ. IKK activation relies on the phosphorylation of IKKα at Ser-176 and IKKβ at Ser-177. Active IKK phosphorylates IκB at Ser-32/36^10^, which is subsequently degraded^11^.

Protein tyrosine phosphatases (PTPs) have been reported to act as key regulators of immune responses by regulating the MAPK pathway^12^. Through inactivation of the c-Jun N-terminal kinase (JNK) and p38 pathways, mitogen-activated protein kinase phosphatase-1 (MKP-1), a PTP, negatively regulates pro-inflammatory cytokines in LPS-stimulated RAW 264.7 cells^13^. Our previous studies have shown that other PTPs, including receptor-type tyrosine-protein phosphatase epsilon (PTPRE), MKP-8, PTP non-receptor type 3 (PTPN3), and PTPN7, are involved in anti-inflammatory responses. Their mRNA and protein levels are influenced by LPS treatment and changes in their expression levels reduce TNF-α levels by targeting particular MAPKs such as JNK, p38, or extracellular signal-regulated kinase (ERK) in RAW 264.7 cells^14–17^.

Dual-specificity phosphatase 5 (DUSP5), also called VH1-like phosphatase-3 (VH3), is an MKP^18,19^. DUSP5 expression is induced by either heat shock or growth factor expression in mammalian cells^20^. In addition, sustained inflammation caused by NF-κB activation in irradiated human arteries leads to DUSP5 overexpression^21^. In contrast to other inducible MKPs, including MKP-1/DUSP1, MKP-2/DUSP4, and PAC1/DUSP2, that interact with and inactivate both mitogen- and stress-activated MAPKs, DUSP5 is highly selective in its ability to bind and inactivate ERK1 and ERK2 *in vivo* ^22^. Furthermore, DUSP5 is a direct transcriptional target of the tumor suppressor p53 and has tumor-suppressive functions in several types of cancer^23,24^.

Since other DUSPs, such as DUSP4 and DUSP6, which also inactivate ERKs, failed to show tumor suppression in gastric cancer^23^, it will be interesting to identify the novel targets of DUSP5 and investigate their functional relationships. In the present study, we show that DUSP5 inhibits NF-κB signal transduction. DUSP5 expression increases in RAW 264.7 cells following exposure to LPS. Overexpression or knockdown of DUSP5 regulates the production of pro-inflammatory cytokines TNF-α and IL-6 by regulating the phosphorylation of ERK1/2 and NF-κB in LPS-stimulated RAW 264.7 cells. Interestingly, DUSP5 differentially regulates these signals. DUSP5 phosphatase activity is required for ERK regulation, whereas DUSP5 regulates NF-κB activity in a phosphatase activity-independent manner by interfering with the association between TAK1 and IKKs, which are upstream factors in NF-κB signal transduction. The inhibitory role of DUSP5 on NF-κB signaling was also confirmed using *DUSP5* knockout (KO) cells.

## Results

### DUSP5 expression is transiently induced by LPS in macrophages

**Figure 1:**
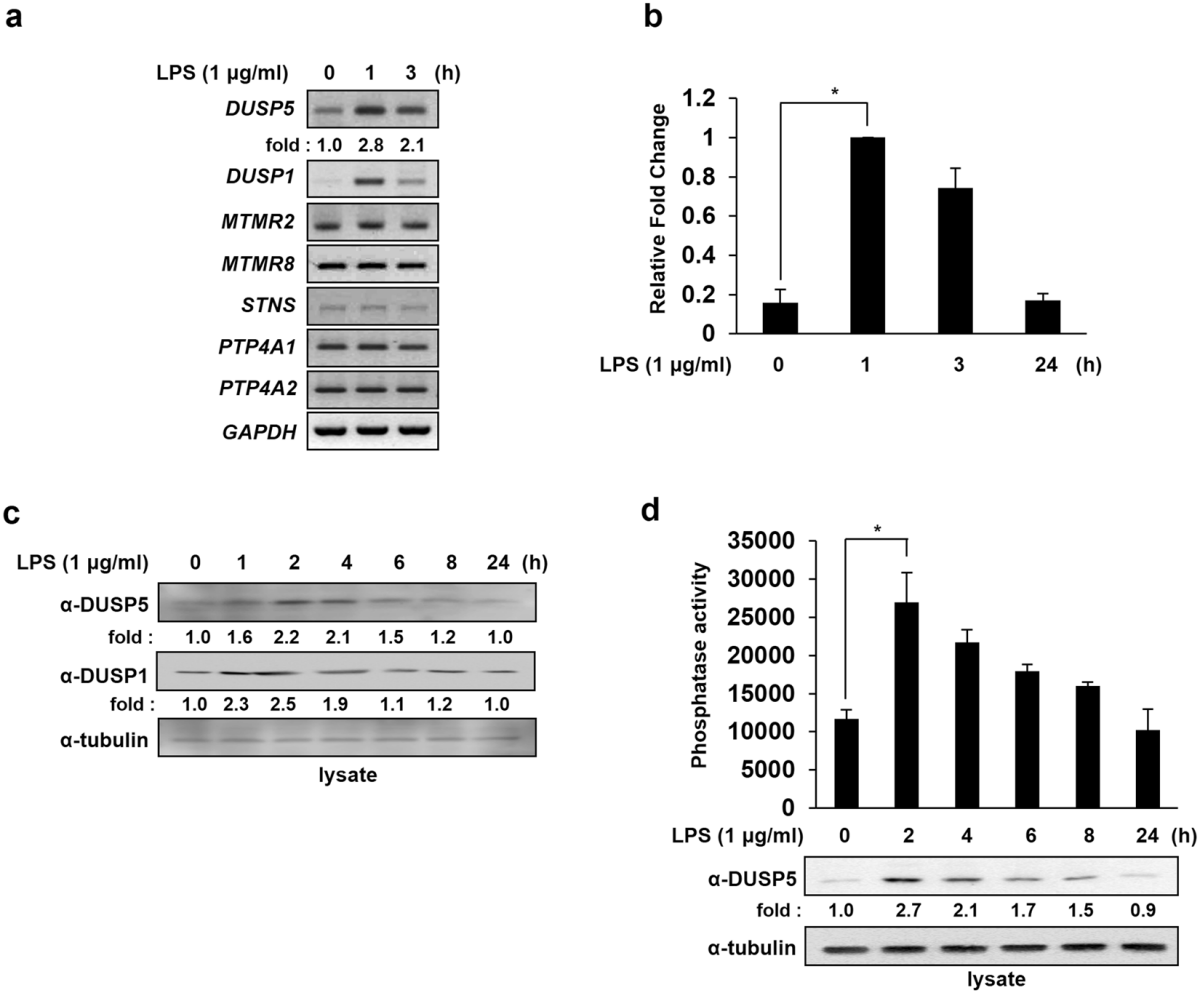
DUSP5 is transiently induced following LPS treatment in RAW 264.7 cells. (**a**) cDNA was synthesized from total RNA extracted from RAW 264.7 cells at the indicated time points after treatment with LPS (1 μg/ml). PTP transcripts were amplified using specific primers (listed in Table 1). GAPDH transcripts amplified using specific primers (forward 5′-ACCACCATGGAGAAGGC-3′; reverse 5′-CTCAGTGTAGCCCAGGATGC-3′) were used as controls. Similar results were obtained in three independent experiments. (**b**) *DUSP5* mRNA expression levels in RAW 264.7 cells were determined at the indicated time points after LPS treatment (1 μg/ml) by quantitative real-time PCR. GAPDH transcripts amplified using specific primers were used as controls. Relative mRNA expression levels were normalized to GAPDH and are presented as fold increases. The results represent mean data from three independent experiments. *p < 0.05, compared with untreated samples (Student’s *t*-test). (**c**) DUSP5 protein expression in RAW 264.7 cells was analyzed at various time points after LPS stimulation (1 μg/ml) by immunoblotting with the appropriate antibodies. The experiments were repeated thrice, and yielded similar results each time. Protein expression levels were quantified by scanning the immunoblots and analyzing the scans with LabWorks software. The relative expression levels of DUSP5 and DUSP1 were normalized to the expression level of tubulin and are presented as fold increases. (**d**) RAW 264.7 cells were untreated or treated with 1 μg/ml of LPS for 2, 4, 6, 8, or 24 h. The cells were harvested and immunoprecipitated with anti-DUSP5 antibody. *In vitro* phosphatase assays were performed as described in Materials and Methods. The results presented represent mean data from three independent experiments. *p < 0.05 versus untreated samples (Student’s *t*-test). Protein expression levels were analyzed by immunoblotting and quantified by scanning the immunoblots and analyzing the scans using LabWorks software.

Several signaling pathways in innate immune cells are activated by a protein phosphorylation cascade that leads to synthesis of pro-inflammatory cytokines that mobilize the immune system to combat LPS, endotoxins derived from pathogenic gram-negative bacteria^25^. During inflammation, phosphorylation of signaling components is regulated by phosphatases induced by LPS stimulation^26,27^. Since several PTPs are induced or suppressed by LPS in order to control protein phosphorylation during inflammation in macrophages^12,14–17^, we performed RT-PCR with RNA samples prepared from RAW 264.7 cells stimulated with LPS for 1 or 3 h, using gene-specific primers against previously untested PTP genes (Table 1). *DUSP1* primers were used as a positive control, since *DUSP1* expression is known to be induced by LPS^28^. Of the PTP genes tested, *DUSP5* expression increased upon exposure to LPS, whereas the expression of the other PTPs did not change (Fig. 1a). The list of PTPs analyzed in this study and in previous studies is shown in Table 2. *DUSP5* mRNA expression was induced within 1 h of LPS stimulation, which then began to decline by 3 h of LPS stimulation. Using quantitative real-time polymerase chain reaction (qRT-PCR), we confirmed the kinetics of *DUSP5* mRNA expression changes after LPS treatment. *DUSP5* mRNA expression increased when the cells were treated with LPS for 1 h and then slowly returned to near-basal levels by 24 h (Fig. 1b). In addition, when DUSP5 protein expression levels were analyzed by immunoblotting using an anti-DUSP5 antibody, the protein expression pattern corresponded to the mRNA expression pattern, although it was slightly delayed (Fig. 1c). Similar to DUSP5, DUSP1 was induced at early time points following LPS exposure and then returned to basal levels after 2 h of LPS treatment, as reported previously^28^. Endogenous DUSP5 was immunoprecipitated from LPS-treated RAW 264.7 and its phosphatase activities were measured using OMFP as a substrate. DUSP5 phosphatase activity was found to be increased in proportion to the increase in its protein expression (Fig. 1d). These results suggest that DUSP5 expression and its activity are induced by LPS treatment at an early stage.

**Table 1:** Primer sequences for PTP genes amplified by RT-PCR.

Gene	Accession number	Primer used for PCR analysis
*DUSP5*	XM_045322	Forward: 5′-GAGGCAAGGTCCTGGTCCAC-3′ Reverse: 5′-GCCTCCCCTTGGCAGGAG-3′
*MKP1*	XM_003720	Forward: 5′-CCTGTGGAGGACAACCACAAGG-3′ Reverse: 5′-GCTGGCCCATGAAGCTGAAG-3′
*MTMR2*	XM_045710	Forward: 5′-GGCCATGGAGATAAGAACCATGC-3′ Reverse: 5′-GCGCATGCTGGCTACTGG-3′
*MTMR8*	BC012399	Forward: 5′-CGGTGATCGTGGGCAGTTC-3′ Reverse: 5′-CATTGAGCTTGGGCCTGGTATC-3′
*PTP4A1*	NM_003463	Forward: 5′-CCTGGTTGCTGTATTGCTGTCCATTG-3′Reverse: 5′-CAGGCACCCCAGTTTTATTGAATACAAC-3′
*PTP4A2*	NM_003479	Forward: 5′-GGAATCCACGTTCTAGATTGGCC-3′ Reverse: 5′-GCCCATTGGTATCTCTGAAGCG-3′
*STNS*	XM_058659.5	Forward: 5′-CCCTGGGACCATGGAGGAC-3′ Reverse: 5′-GTCAGCCGTTCTGTGCGG-3′

**Table 2:** The list of PTPs analyzed in response to LPS.

Gene	Induction by LPS treatment	References
DUSP5	Ind	This study
MKP1	Ind	This study, ^47^
DUSP2	Ind	^48^
DUSP3	No effect	^49^
DUSP4	Ind	^50^
DUSP6	No effect	^49^
DUSP7	No effect	^14^
DUSP10	Ind	^51^
DUSP11	No effect	^14^
DUSP12	No effect	^14^
DUSP14	No effect	^49^
DUSP18	No effect	^14^
DUSP22	No effect	^49^
DUSP26	No effect	^16^
PTPRE	No effect	^15^
PTPN2	No effect	^14^
PTPN3	Ind	^17^
PTPN7	Red	^14^
PTPN18	No effect	^14^
PTP4A1	No effect	This study
PTP4A2	No effect	This study
MTMR2	No effect	This study
MTMR8	No effect	This study
STNS	No effect	This study

Abbreviations: Red, reduced; Ind, induced.

### DUSP5 inhibits TNF-α and IL-6 production

**Figure 2:**
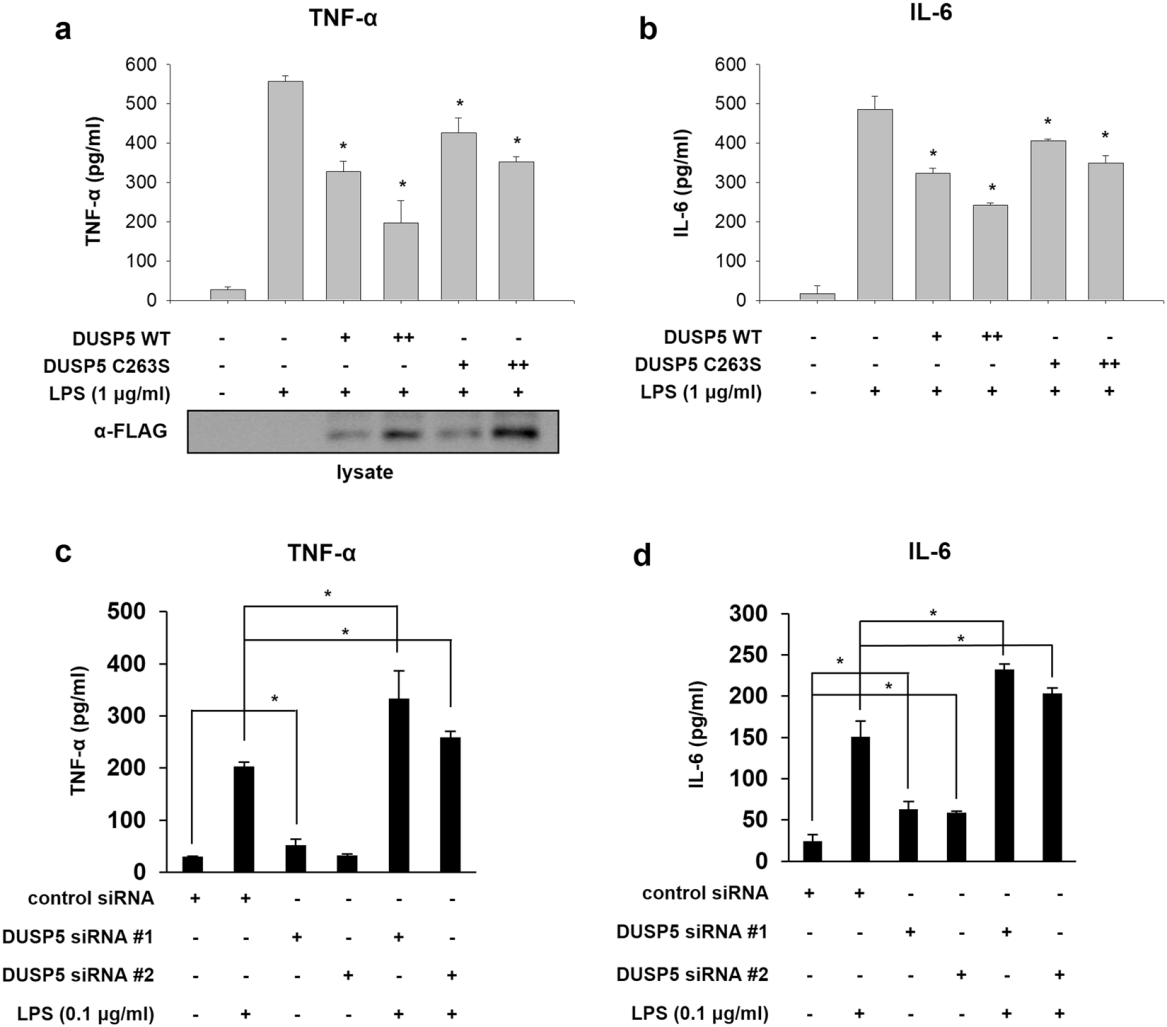
DUSP5 inhibits LPS-stimulated cytokine production in RAW 264.7 cells RAW 264.7 cells were transfected with either FLAG-DUSP5-WT or -C263S. After 16 h stimulation with LPS (1 μg/ml), the levels of TNF-α (**a**) and IL-6 (**b**) in the supernatants were analyzed by ELISA, as described in Materials and Methods. Cell lysates were subjected to immunoblotting using an anti-FLAG antibody for the detection of DUSP5. The results represent three independent experiments. *p < 0.05 versus LPS-treated cells transfected with empty vector (Student’s *t*-test). After transfection with control or *DUSP5* siRNAs (#1 and #2), cells were treated with 0.1 μg/ml LPS for 1 h, and the levels of TNF-α (**c**) and IL-6 (**d**) were measured by ELISA. The results represent mean data from three independent experiments. *p < 0.05 versus LPS-treated or untreated cells transfected with control siRNA (Student’s *t*-test).

Since LPS induces DUSP5 expression in RAW 264.7 cells, DUSP5 might be involved in the regulation of pro-inflammatory cytokines. To investigate the effect of DUSP5 on cytokine production, we first analyzed the levels of TNF-α and IL-6, which are major inflammatory cytokines induced in macrophages upon exposure to endotoxins such as LPS^29,30^. To examine the effect of DUSP5 on TNF-α and IL-6 production, RAW 264.7 cells were transfected with a mammalian expression vector containing FLAG-tagged DUSP5 wild type (WT) or the catalytically inactive C263S mutant for 32 h and then stimulated with 1 μg/ml LPS for 16 h prior to assessment of TNF-α and IL-6 production by ELISA. Compared to LPS-treated cells, both DUSP5 WT- and C263S mutant-expressing cells produced reduced levels of TNF-α and IL-6 (Fig. 2a and b). However, the inhibitory effect of DUSP5 C263S mutant on cytokine production was weaker than that of DUSP5 WT, which suggests that DUSP5 regulates both TNF-α and IL-6 in a phosphatase activity-dependent and -independent manner. To further confirm that the production of TNF-α and IL-6 is regulated by DUSP5, *DUSP5*-specific siRNAs were transfected into RAW 264.7 cells to knock down *DUSP5* gene expression. Reduced levels of DUSP5 expression after transfection with *DUSP5*-specific siRNAs (#1 and #2) were confirmed by immunoblotting **(**Supplementary Figure 1). Following transfection with either scrambled control siRNA or *DUSP5*-specific siRNAs (#1 and #2), RAW 264.7 cells were treated with a low dose of LPS (0.1 μg/ml) for 1 h to avoid saturation of TNF-α and IL-6 production, and the effects of *DUSP5* knockdown were then determined by measuring the TNF-α and IL-6 levels in the growth medium. As shown in Fig. 2c,d, *DUSP5* knockdown enhanced LPS-induced TNF-α and IL-6 production in RAW 264.7 cells. Furthermore, TNF-α and IL-6 levels were increased in the absence of LPS when *DUSP5* was knocked down. Taken together, these results suggest that DUSP5 inhibits the production of TNF-α and IL-6 in RAW 264.7 macrophages.

### DUSP5 regulates NF-κB as well as ERK1/ 2 signal transduction

**Figure 3:**
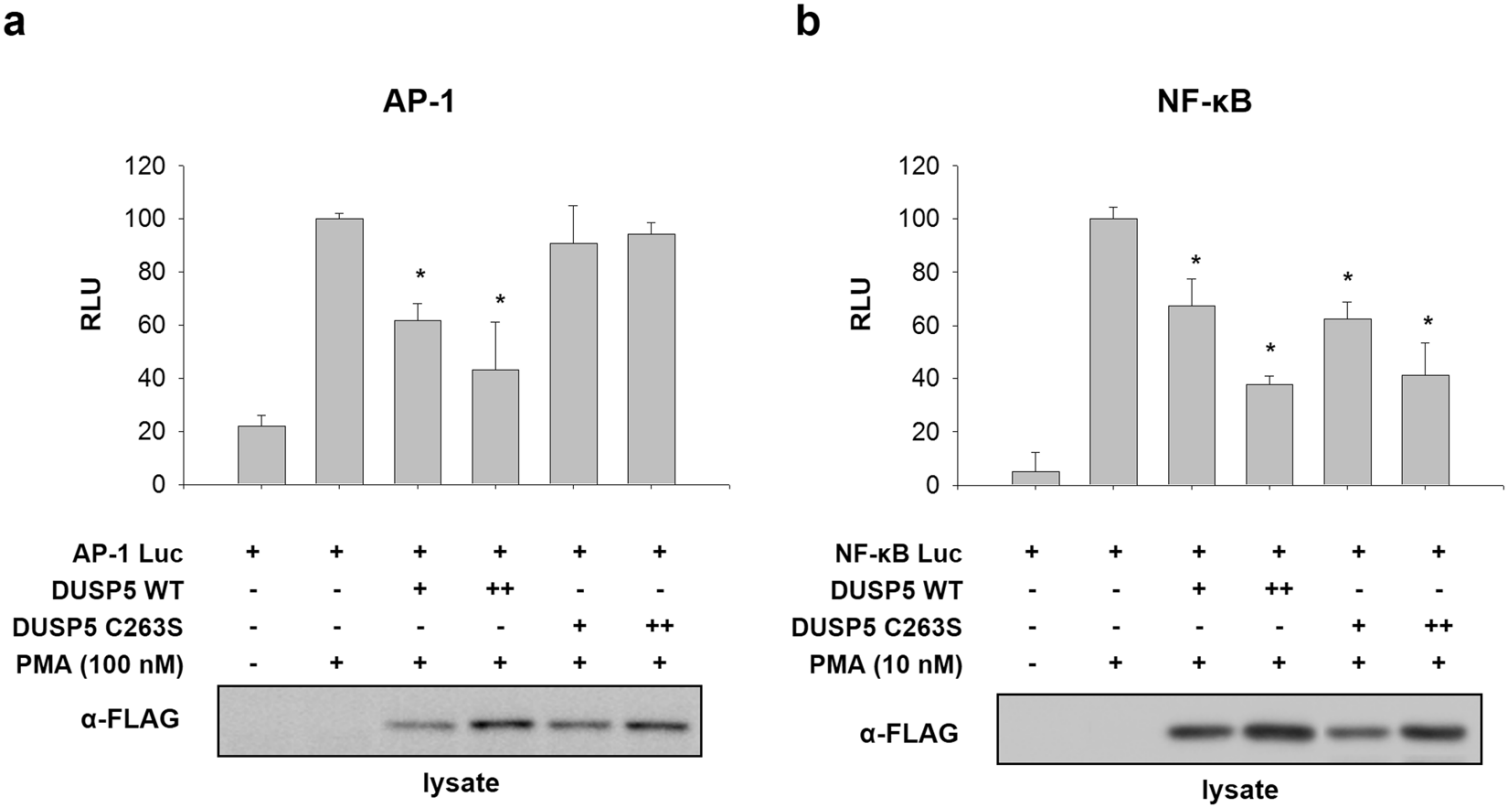
DUSP5 expression regulates AP-1 and NF-κB transcriptional activity. HEK 293 cells were transiently co-transfected with the reporter plasmids AP-1-Luc (**a**) or NF-κB-Luc (**b**) and gWIZ-GFP, together with either FLAG-DUSP5 WT or C263S, for 32 h. Cell were treated with the indicated concentrations of PMA (100 nM for AP-1-Luc and 10 nM for NF-κB-Luc) for an additional 16 h. Luciferase activity was normalized to GFP activity. Data are expressed as relative fold increase in luciferase units (RLU) compared to the PMA-treated group (100%). All data represent the mean of three independent experiments *p < 0.05 versus PMA-treated cells transfected with empty vector (Student’s *t*-test).

Pro-inflammatory cytokines are regulated by two major signaling pathways, MAPK and NF-κB. During inflammation, inflammatory gene expression is induced by the activation of specific transcription factors such as AP-1 and NF-κB. The signal transduction cascade after pathogenic stimulation results in the activation of parallel kinase cascades that regulate AP-1 and NF-κB^25^. We therefore carried out luciferase activity-based reporter assays to investigate whether DUSP5 regulates AP-1 and NF-κB transcription activity. An AP-1- or NF-κB-Luc reporter plasmid was co-transfected with the FLAG-tagged DUSP5 WT or C263S plasmid into HEK 293 cells. Transfected cells were then treated with PMA to stimulate AP-1 and NF-κB activity. The PMA treatment was sufficient for stimulating the reporter genes and transient expression of DUSP5 WT resulted in decreased transcriptional activity of AP-1 and NF-κB in a dose-dependent manner (Fig. 3a,b). Interestingly, DUSP5 C263S inhibited the PMA-induced NF-κB transcriptional activity, whereas the mutant failed to inhibit AP-1 activity, suggesting that DUSP5 regulates NF-κB transcription in a phosphatase activity-independent manner, whereas regulation of AP-1 by DUSP5 is dependent on its phosphatase activity (Fig. 3b), which is reminiscent of the data on the regulation of TNF-α and IL-6 production by DUSP5 C263S mutant, as shown in Fig. 2a and b.

### DUSP5 regulates NF-κB signal transduction independently of its phosphatase activity

Since DUSP5 inhibits the transcriptional activity of AP-1 and NF-κB, we examined the effect of DUSP5 on MAPK and NF-κB signaling in LPS-stimulated RAW 264.7 cells. DUSP5 has been previously reported to play a role in the regulation of MAPKs^20^. DUSP5 is known to specifically dephosphorylate the TXY motifs of ERK1/2^20^. We confirmed that DUSP5 dephosphorylates the TXY motifs of ERK1/2 but not p38 or JNK in RAW 264.7 cells using phospho-specific antibodies. Immunoblotting analysis of lysates from RAW 264.7 cells transfected with FLAG-tagged DUSP5 WT or C263S plasmids followed by stimulation with LPS showed that the endogenous phospho (p)-ERK1/2 level was markedly reduced in DUSP5 WT-transfected cells compared to that in cells transfected with the C263S mutant (Supplementary Figure 2). Under the same conditions, the levels of phosphorylated JNK and p38 were unchanged.

**Figure 4:**
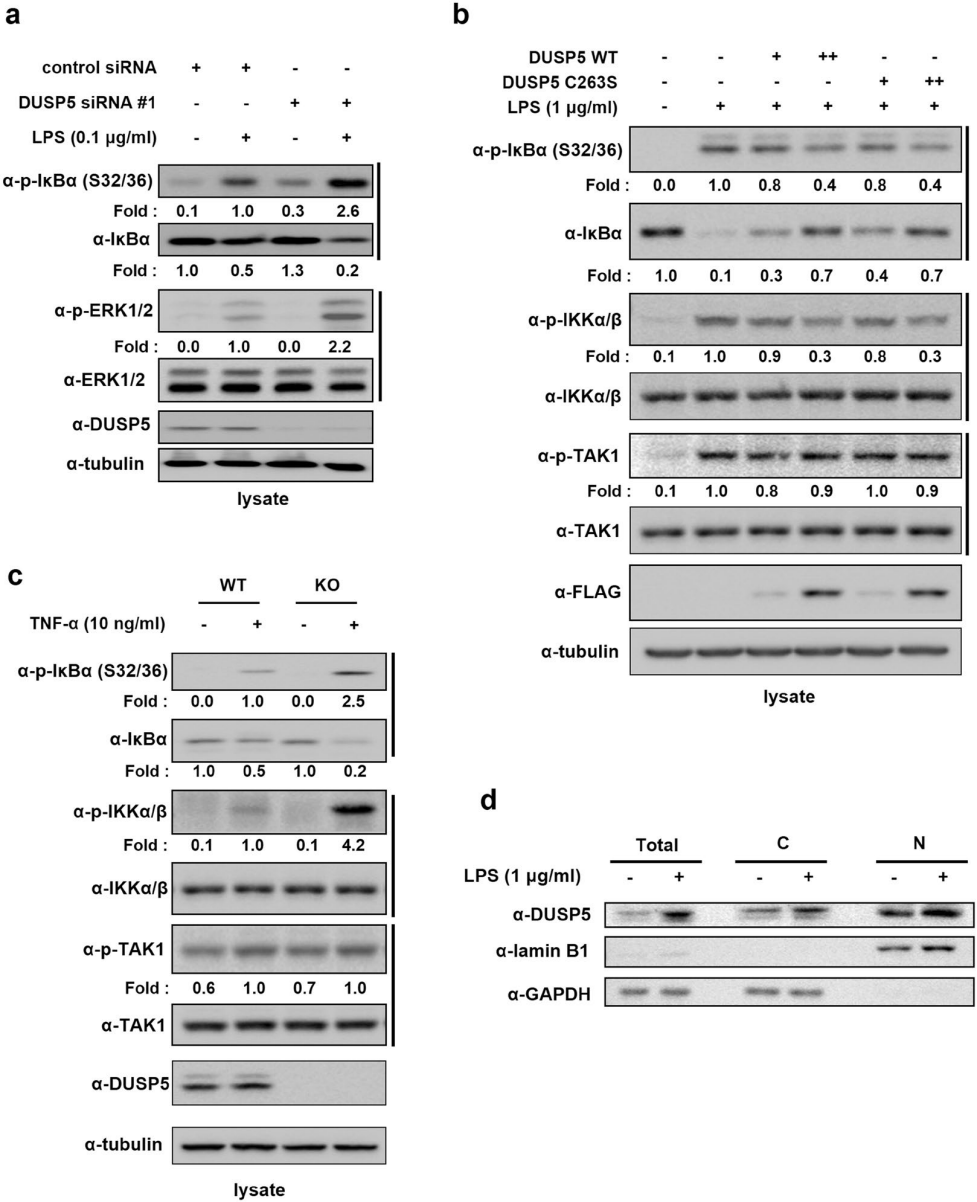
Effect of DUSP5 on LPS-mediated MAPK and NF-κB signal transduction (**a**) RAW 264.7 cells were transfected with control siRNA or *DUSP5* siRNA #1 for 48 h and were stimulated with LPS (0.1 μg/ml) for 30 min. ERK1/2 and IκBα phosphorylation levels were determined by immunoblotting with the appropriate antibodies. The phosphorylation levels of ERK1/2 were normalized to the corresponding total ERK1/2 levels, whereas those of p-IκBα and total IκBα were normalized to tubulin levels; all data are presented as fold increases. Similar results were obtained in three independent experiments. (**b**) DUSP5 WT- or C263S-transfected RAW 264.7 cells were stimulated with LPS (1 μg/ml) for 15 min. Immunoblots were probed with the indicated antibodies. The levels of p-IκBα and total IκBα were normalized to tubulin expression levels and presented as fold increases. The levels of p-IKKα/β and p-TAK1 were normalized to the expression levels of IKKα/β and TAK1. Similar results were obtained in three independent experiments. (**c**) *DUSP5* WT and KO MEF cells were treated with TNF-α for 10 min and total cell lysates were then obtained. The same quantity of total proteins was subjected to immunoblotting analysis with appropriate antibodies. The levels of p-IκBα and total IκBα were normalized to tubulin expression levels. The levels of p-IKKα/β and p-TAK1 were normalized to the expression levels of IKKα/β and TAK1. The relative levels of p-IκBα, total IκBα, p-IKKα/β, and p-TAK1 were presented as fold increases. Similar results were obtained in three independent experiments. (d) RAW 264.7 cells were left untreated or stimulated with 1 μg/ml LPS for 2 h. Harvested cells were lysed (Total) or fractionated into cytoplasmic (**C**) and nuclear (N) fractions. Each fraction (30 μg for total cell lysate, 40 μg for cytoplasmic or nuclear fraction) was immunoblotted using specific antibodies. Anti-GAPDH and anti-Lamin B1 antibodies were used to verify the efficient fractionation of cytoplasmic and nuclear proteins, respectively. Similar results were obtained in three independent experiments.

To confirm that DUSP5 expression affects the phosphorylation of IκBα, *DUSP5* was knocked down by transfection with *DUSP5* siRNA #1 into RAW 264.7 cells. *DUSP5* knockdown enhanced the phosphorylation of IκBα at Ser-32/36 and degradation of IκBα, compared to that in cells transfected with the non-targeting control siRNA (Fig. 4a). ERK1/2 phosphorylation was enhanced by *DUSP5* knockdown, as expected. These results suggest that DUSP5 regulates the levels of p-IκBα as well as p-ERK1/2 in LPS-stimulated RAW 264.7 cells.

Unlike ERK1/2, NF-κB has not been reported as a target of DUSP5. Therefore, we investigated how DUSP5 regulates NF-κB activity. Since phosphorylation of IκBα at Ser-32/36 is the most well-known process leading to ubiquitin-mediated degradation of IκBα and release of NF-κB for nuclear translocation^31,32^, we examined the levels of p-IκBα (Ser-32/36) in *DUSP5*-transfected cells by immunoblotting analysis. Similar to the result in Fig. 3b, both DUSP5 WT and C263S reduced the phosphorylation levels of IκBα Ser-32/36 and inhibited the degradation of IκBα in a dose-dependent manner (Fig. 4b). To clarify the mechanism of action of DUSP5 on regulation of NF-κB signal transduction, the phosphorylation levels of signaling kinases upstream of IκBα, including IKKα/β and TAK1, in the presence of DUSP5 was investigated. As shown in Fig. 4b, phosphorylation at Ser-176/180 of IKKα/β, a kinase complex directly upstream of IκBα, was also decreased by DUSP5 WT or C263S expression. However, *DUSP5* WT- and C263S-transfected cells did not result in altered phosphorylation levels at Thr-184/187 of TAK1 that phosphorylates and activates IKKs. These results imply that DUSP5 might regulate the NF-κB signaling pathway by acting on TAK1 independently of phosphatase activity.

The regulatory roles of DUSP5 in the NF-κB signaling pathway were confirmed by employing *DUSP5* WT and KO mouse embryonic fibroblasts (MEFs). We examined the phosphorylation levels of NF-κB signal transduction molecules, including IκBα, IKKα/β, and TAK1 (Fig. 4c). Treatment with LPS for stimulation of NF-κB signal transduction did not result in any response from the cells. Therefore, MEFs were treated with TNF-α to stimulate NF-κB signal transduction because TNF-α-induced NF-κB activation is mediated by activation of the TAK1/IKK/IκBα axis. The phosphorylation levels of IκBα, IKKα/β, and TAK1 were enhanced by TNF-α stimulation in both *DUSP5* WT and KO cells. However, *DUSP5* KO cells showed a greater increase in the fold induction of IκBα and IKKα/β phosphorylation than that in the *DUSP5* WT cells. IκBα levels decreased due to degradation of phosphorylated IκBα in *DUSP5* KO cells upon TNF-α stimulation, implying that DUSP5 expression is critical for the activation of NF-κB signal transduction via inhibition of IKKα/β and subsequent IκBα phosphorylation and degradation. However, no difference in TNF-α-induced phosphorylation of TAK1 between *DUSP5* WT and KO cells was obvious. These results suggest that DUSP5 expression is critical for the negative regulation of NF-κB signal transduction by either dephosphorylating IKKα/β or by physically inhibiting TAK1.

Since our data suggest that DUSP5 acts as a regulator of NF-κB signaling in the cytoplasm, we next investigated the subcellular localization of endogenous DUSP5 in LPS-stimulated RAW 264.7 cells. As shown in Fig. 4d, DUSP5 expression was detected in both the cytoplasm and the nucleus. In addition, treatment with LPS resulted in significantly increased expression of DUSP5 in both the cytoplasmic and nuclear fractions. Although nuclear localization of DUSP5 has been reported^22^, our data provide evidence that DUSP5 can also exist in the cytoplasm and that DUSP5 expression in both the cytoplasm and the nucleus is upregulated upon LPS stimulation.

### DUSP5 physically interacts with TAK1 and IKKα/β and blocks the association of TAK1 with IKKα/β

**Figure 5:**
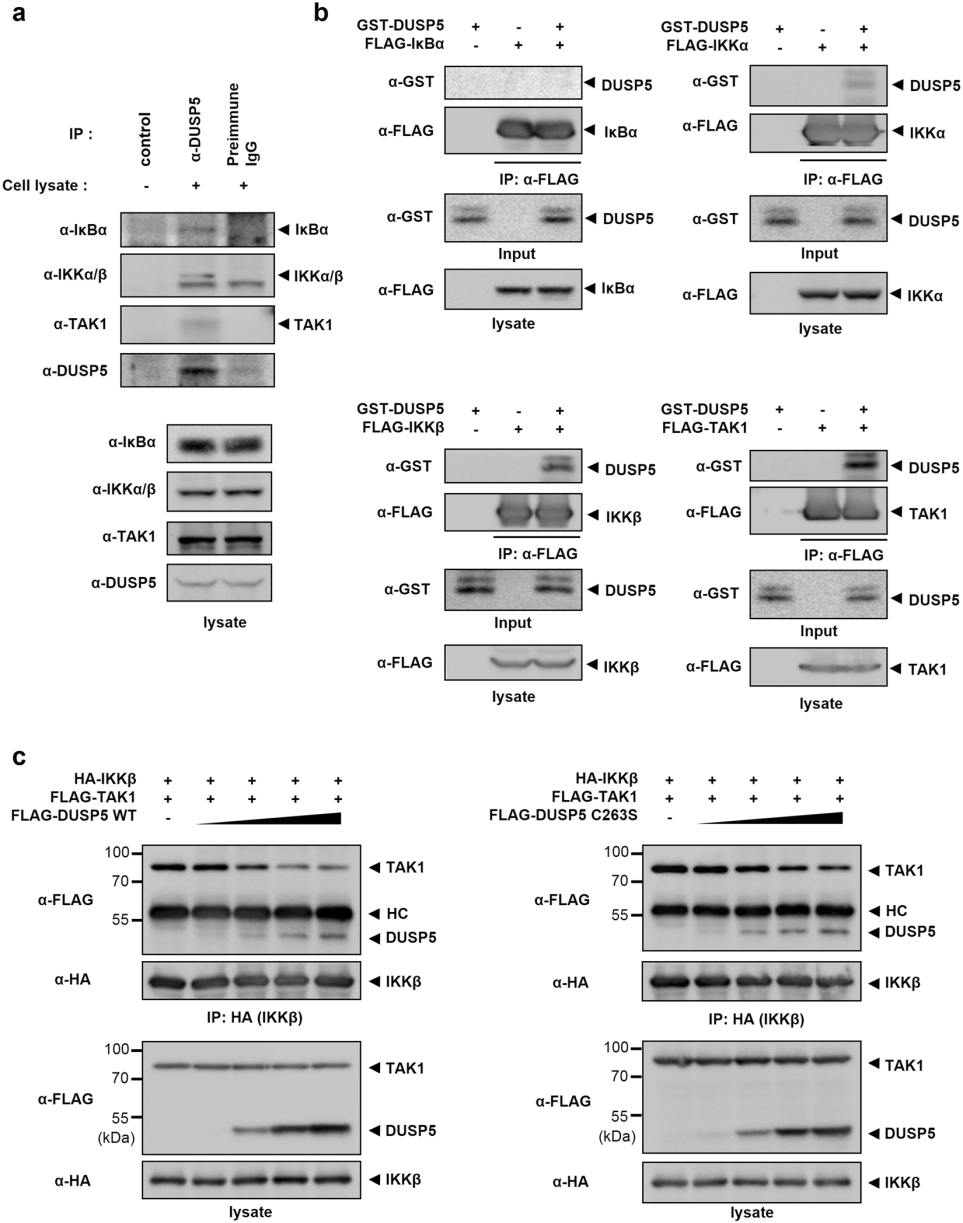
DUSP5 directly interacts with IKKα/β and TAK1 to regulate the NF-κB signaling pathway. (**a**) Cell lysates from LPS-treated RAW 264.7 were immunoprecipitated with goat pre-immune IgG or anti-DUSP5 antibody, and then incubated with protein A/G beads. Bound proteins were identified with anti-IκBα, anti-IKKα/β, and anti-TAK1 antibodies. The far left lane (control) shows anti-DUSP5 antibody incubated with protein A/G in the absence of cell lysates to confirm the lack of indigenous IgG reactivity. IP. immunoprecipitation. (**b**) FLAG-tagged IκBα, IKKα/β, or TAK1 were purified from transfected HEK 293 cells using anti-FLAG M2 agarose and the anti-FLAG bead-bound proteins were then mixed with recombinant GST-DUSP5 (2 μg). After binding, the bead-bound proteins were subjected to immunoblotting analyses with the indicated antibodies to detect a direct interaction between FLAG-tagged proteins and GST-DUSP5. (**c**) HEK 293 cells co-transfected with HA-IKKβ and FLAG-TAK1 expression plasmids were then transfected with various doses (0.5, 1.0, 1.5, and 2.0 μg) of FLAG-DUSP5 WT or C263S expression plasmids. Total cell lysates were incubated with anti-HA antibody, then immunoprecipitated with protein A/G beads. Bound proteins were identified by immunoblotting analyses using an anti-FLAG antibody. Similar results were obtained in three independent experiments. HC. immunoglobulin heavy chain.

As described above, DUSP5 inhibits the phosphorylation of IκBα and IKKα/β in a phosphatase activity-independent manner. Furthermore, DUSP5 had no effect on TAK1 phosphorylation. These data led us to examine the regulatory mechanism of DUSP5 by investigating the interaction partners of DUSP5 in the NF-κB signaling pathway. Since endogenous DUSP5 was induced upon LPS stimulation in RAW 264.7 cells, we carried out co-immunoprecipitation using either pre-immune IgG or anti-DUSP5 antibody with total cell lysates obtained from LPS-stimulated RAW 264.7 cells to determine the NF-κB signaling proteins associated with DUSP5. As shown in Fig. 5a and Supplementary Figure 3, IκBα, IKKα/β, and TAK1 were detected in immunoprecipitated DUSP5 complexes from RAW 264.7 cell lysates but not in immunoprecipitated pre-immune IgG complexes. We also confirmed that DUSP5 associates with TAK1, IKKα/β, and IκBα, in *DUSP5* WT MEFs whereas no interaction was detected in *DUSP5* KO cells (Supplementary Figure 4). These results imply that the NF-κB signaling pathway is regulated by the formation of a molecular complex between DUSP5 and NF-κB signal transduction molecules, including IκBα, IKKα/β, and TAK1.

However, the data from the co-immunoprecipitation between DUSP5 and endogenous NF-κB signaling molecules do not explain the fact that DUSP5 is not involved in the regulation of TAK1 phosphorylation even though DUSP5 and TAK1 associate in cells. We, therefore, carried out *in vitro* binding assays to identify the direct interacting partners of DUSP5. FLAG-IκBα, -IKKα, -IKKβ, or -TAK1 proteins were purified from transfected cells by extensive washing after binding of FLAG-fused proteins to anti-FLAG M2 agarose and then incubated with purified recombinant GST-DUSP5. As shown in Fig. 5b, FLAG-IKKα, FLAG-IKKβ, and FLAG-TAK1 proteins interacted with GST-DUSP5 *in vitro*. However, GST-DUSP5 failed to interact with FLAG-IκBα. These results indicate that DUSP5-mediated dephosphorylation of p-IκBα is not due to a direct interaction between DUSP5 and IκBα.

These results led to a hypothesis that DUSP5-mediated regulation of NF-κB signal transduction might be conducted by the physical intervention of DUSP5 between IKKα/β and TAK1, and thereby inhibition of IKKα/β phosphorylation. We therefore investigated whether DUSP5 inhibits IKK binding to TAK1 in a dose-dependent manner in cells. Total cell lysates from cells transfected with HA-IKKβ, FLAG-TAK1, and increasing amounts of FLAG-DUSP5 WT or C263S expression plasmids, were subjected to immunoprecipitation using anti-HA antibodies. After removal of unbound proteins, the levels of DUSP5 and TAK1 bound to IKKβ were detected by immunoblotting analysis. With increasing DUSP5 WT or C263S, the levels of TAK1 bound to IKKβ were gradually reduced in a dose-dependent manner (Fig. 5c). Furthermore, DUSP5 WT failed to directly dephosphorylate p-IκBα and p-IKKα/β *in vitro* (Supplementary figure 5). These results indicate that DUSP5 acts as a competitor of TAK1 for binding to IKKβ and that its phosphatase activity is not necessary for this competition.

## Discussion

DUSP5 is a potent pro-inflammatory regulator induced by IL-2, IL-7, and IL-15, and inhibits IL-2-induced ERK1/2 activation^33,34^. In this report, we showed that DUSP5 was transiently induced during LPS-mediated inflammatory responses in RAW 264.7 macrophages. In addition, transient *DUSP5* overexpression suppressed TNF-α and IL-6 production through inactivation of both ERK1/2 and NF-κB pathways. Both the MAPK (ERK, JNK, and p38) and NF-κB signaling pathways are reportedly activated in RAW 264.7 macrophages upon exposure to LPS^35–37^. It has also been reported that DUSP5 expression is regulated mainly at the transcriptional level by the transcription factor Elk-1^38^, and that Elk-1 is phosphorylated and thus activated by ERK^39^, suggesting that LPS induces ERK-mediated Elk-1 activation and thus DUSP5 activity. Furthermore, sustained inflammation caused by NF-κB activation induced DUSP5 expression in irradiated human arteries^21^. These results imply that the transient induction of DUSP5 is involved in negative feedback regulation during the LPS-induced inflammatory response via inactivation of both ERK1/2 and NF-κB pathways. Since DUSP5 acts as a negative regulator of both ERK1/2 and NF-κB signal transduction in macrophages, the role of DUSP5 on signal transduction in the regulation of inflammatory responses may be more important than that of any other phosphatase.

That DUSP5 regulates NF-κB signal transduction in macrophages is a novel finding. In a previous genome-wide study, it was shown that knockdown of *DUSP5* increased NF-κB activity, but the mechanism underlying the finding was not investigated^40^. DUSP5 failed to dephosphorylate p-IκB and several NF-κB–regulating phosphatases are known to act on NF-κB or other upstream kinases such as IKK or TRAF2 but not on IκB^41^, suggesting that DUSP5 might target upstream kinases. Interestingly, previous reports showed that DUSP5 is localized in the nucleus and regulates nuclear ERK activity^22,42^, but NF-κB upstream kinases are localized in the cytoplasm, which causes a subcellular localization conflict. However, our data and another report^43^ show that DUSP5 is localized in both the cytoplasmic and nuclear fractions, suggesting the possibility of a physical association between DUSP5 and the NF-κB upstream kinases.

NF-κB activity might be regulated through the action of DUSP5 as a scaffold since DUSP5 interacts with both TAK1 and IKKs. In addition, DUSP proteins that act as scaffold proteins do not require their phosphatase activities^44,45^, which is the characteristic of DUSP5 for the regulation of NF-κB signaling. If DUSP5 acts as a scaffold in NF-κB signaling, DUSP5 should facilitate the association between TAK1 and IKKs. However, as shown in Fig. 5c, increase in DUSP5 concentration resulted in decreased association between the two proteins in a dose-dependent manner regardless of DUSP5 phosphatase activity, indicating that DUSP5 is not a scaffold protein.

Recent reports have proposed that DUSP5 may act as a tumor suppressor by regulating ERK activity in several types of cancer^23,24,43^. However, it is conceivable that other *in vivo* substrates of DUSP5 are involved in its role as a tumor suppressor, since at least 10 different DUSP proteins have the ability to dephosphorylate ERKs^12^. Likewise, DUSP5 might also affect multiple functions in several different cellular responses, including inflammation and tumor suppression. Therefore, it is necessary to investigate the substrate specificity of the DUSP family of proteins, their individual specificities for various signaling kinases, tissue expression specificity, subcellular localization, and induction. On the basis of our findings, we suggest that DUSP5 might have a beneficial effect against several immune diseases by inhibiting ERK1/2 and NF-κB signal transduction in the macrophage inflammatory response cascade.

## Materials and Methods

### Cell culture and Transfection

Mouse macrophage-like RAW 264.7 and human embryonic kidney (HEK) 293 cells were maintained at 37 °C in Dulbecco’s modified Eagle’s medium (DMEM, Invitrogen, Carlsbad, CA) supplemented with 10% fetal bovine serum (FBS, Invitrogen) and penicillin/streptomycin in the presence of 5% CO_2_. *DUSP5* WT and KO MEF cells were obtained from Dr. Stephen Keyse. For transient transfection, 1.4 × 10^6^ cells were plated in each 60-mm cell culture plate, grown overnight, and transfected with DNA using the OmicsFect (Omics Biotechnology, Taiwan) or Neon Transfection System (Invitrogen).

### Plasmid construction

The FLAG-tagged DUSP5 (WT and C263S mutant), IκBα, IKKα, IKKβ, and TAK1 mammalian expression plasmids were constructed in the pcDNA3.1/Zeo plasmid (Invitrogen). All of the constructs were confirmed by DNA sequencing. The GST- DUSP5 WT and C263S bacterial expression plasmids were constructed in pGEX-6P-1.

### Reagents and Antibodies

Anti-ERK1/2 (cat. #4695, rabbit monoclonal), anti-p-ERK1/2 (Thr-202/Tyr-204) (cat. #9106, mouse monoclonal), anti-p-JNK (Thr-183/Tyr-185) (cat. #4668, rabbit monoclonal), anti-p-p38 (Thr-180/Tyr-182) (cat. #9211, rabbit polyclonal), anti-p-IκBα (Ser-32/36) (cat. #9246, mouse monoclonal), anti-p-IKKα/β (Ser-176/180) (cat. #2697, rabbit monoclonal), anti-p-TAK1 (Thr-184/187) (cat. #4508, rabbit monoclonal), anti-TAK1 (cat. #5206, rabbit monoclonal), anti-Lamin B1 (cat. #12586, rabbit monoclonal) antibodies were from Cell Signaling Technology (Danvers, MA). Anti-DUSP5 (cat. #sc-46926, goat polyclonal), anti-JNK (cat. #sc-7345, mouse monoclonal), anti-p38 (cat. #sc-7921, mouse monoclonal), anti-IKKα/β (cat. #sc-7607, rabbit polyclonal), anti-IκBα (cat. #sc-371, rabbit polyclonal), anti-glyceraldehyde-3-phosphate dehydrogenase (GAPDH, cat. #sc-25778, rabbit polyclonal), and anti-HA (cat. #sc-7392, mouse monoclonal) antibodies were from Santa Cruz Biotechnology (Santa Cruz, CA). Anti-FLAG M2 antibody (cat. #F3165, mouse monoclonal), anti-α-tubulin antibody (cat. #T9206, mouse monoclonal), and LPS were from Sigma-Aldrich (St. Louis, MO). Goat anti-rabbit IgG conjugated to horseradish peroxidase (HRP) (cat. #LF-SA5002) and goat anti-mouse IgG conjugated to HRP (cat. #LF-SA5001) used as second antibodies were obtained from Abfrontier (Seoul, Korea).

### Purification of the GST-tagged proteins

After *E. coli* BL21 (DE3) RIL was transformed with pGEX-6P-1-DUSP5 WT or C263S, GST-DUSP5 expressions were induced with 0.05 mM isopropyl-β-D-thiogalactopyranoside at 16 °C for 20 h. Cells were harvested and then lysed by sonication in 50 mM Tris–HCl (pH 8.0), 500 mM NaCl, 10 mM EDTA, 10 mM EGTA, 10% glycerol, 2 mM phenylmethylsulfonyl fluoride (PMSF), and 5 mM dithiothreitol (DTT). The lysates were clarified at 10,000 rpm for 45 min at 4 °C. The supernatant was applied by gravity flow to a column of glutathione Sepharose 4B beads (PEPTRON, Daejeon, Korea). The resin was washed with wash buffer (50 mM Tris–HCl (pH 7.5), 50 mM NaCl, 5% glycerol, and 1 mM DTT) and then eluted with wash buffer with 20 mM glutathione.

### Subcellular fractionation

The cytoplasmic cell fractionation was carried out as described^46–51^. Briefly, cells were washed twice in ice cold PBS and lysed on ice for 15 min in 200 μl of cytoplasmic lysis buffer without detergent (10 mM HEPES, 60 mM KCl, 1 mM EDTA, 1 mM DTT and 1 mM PMSF) and then 10 μl of 0.075% (v/v) IGEPAL CA-630 (Sigma-Aldrich) was added to disrupt plasma membrane. The cytoplasmic extracts were prepared by brief centrifugation for 10 sec. For the nuclear fraction collection, nuclear pellet was resuspended in 25 μl of nuclear extract buffer (20 mM Tris Cl, 420 mM NaCl, 1.5 mM MgCl_2_, 0.2 mM EDTA, 1 mM PMSF and 25% (v/v) glycerol). The soluble nuclear extracts were isolated by centrifugation. Immunoblotting analysis was performed using anti-GAPDH (cytoplasm) and anti-Lamin B1 (nucleus) antibodies to confirm the cytoplasmic and nuclear extracts.

### *In vitro* phosphatase assays

For *in vitro* phosphatase assays, RAW 264.7 cells were treated with or without LPS and then harvested in PTP lysis buffer (1% IGEPAL CA-630, 0.5% Triton X-100, 150 mM NaCl, 20 mM Tris-HCl (pH 8.0), 1 mM EDTA, 1% glycerol, 1 mM PMSF, and 1 μg/ml aprotinin) for 30 min at 4 °C. Cleared cell lysates from centrifugation were immunoprecipitated with rabbit anti-DUSP5 or anti-IgG antibodies (Santa Cruz) for 3 h at 4 °C followed by incubation with protein A/G agarose for 1 h at 4 °C using rotation device. After incubation, immunoprecipitates were washed three times with PTP lysis buffer and its phosphatase activities were measured using the substrate 3-O-methylfluorescein phosphate (OMFP; Sigma-Aldrich).

### *In vitro* binding assay

HEK 293 cells were transfected with FLAG-tagged IκBα, IKKα, IKKβ, and TAK1 expression plasmid (1 μg) using the OmicsFect for 48 h. Total cell lysates were pulled down with anti-FLAG M2 agarose beads for 3 h and the pulled-down proteins were subjected to extensive washing to purify the FLAG-fusion proteins by excluding any bound proteins in the pulled-down complexes. To determine whether DUSP5 directly bind to IκBα, IKKα, IKKβ, or TAK1, each anti-FLAG bead-bound protein was mixed with GST-DUSP5 WT (2 μg) in 1 ml of PTP reaction buffer (100 mM Tris-HCl (pH 7.5), 40 mM NaCl, and 1 mM DTT) and incubated for 3 h at 4 °C. After incubation, the beads were washed 5 times with binding buffer, 1 X sample buffer was then added and boiled for 5 min at 100 °C. The samples were subjected to immunoblotting analyses using appropriate antibodies.

### Enzyme linked immunosorbent assay (ELISA) of TNF-α and IL-6

TNF-α and IL-6 protein concentrations were determined by sandwich ELISA using antibodies and standards obtained from eBiosciences (San Diego, CA) according to manufacturer’s instructions. Assays were performed on neat and diluted samples in triple on 96-well plates. Absorbance was measured by a microplate reader at 450 nm and concentrations were determined by comparison to a standard curve. All experiments were repeated at least three times.

### Immunoblotting analysis

Immunoblotting was performed with a SDS-PAGE Electrophoresis System as described previously^14^. Briefly, samples were run on SDS-10% polyacrylamide gels and transferred to nitrocellulose membrane. The membranes were blocked in 5% nonfat skim milk and incubated with an appropriate antibody, followed by incubation with a secondary antibody conjugated to horseradish peroxidase. The immunoreactive bands were visualized using an ECL system (Pierce, Rockford, IL) and a cooled charge-device camera system (AE-9150, ATTO Technology, Tokyo, Japan). The intensity of the immunoreactive bands was quantified using LabWorks Analysis software (UVP, LLC, Upland, CA).

### Reverse transcription-polymerase chain reaction (RT-PCR)

Total RNAs were prepared from cells by Accuzol (Bioneer Corporation, Daejon, Korea) and RT was performed using Omniscript RT Kit (Qiagen, Hilden, Germany). PCR for mouse PTPs was carried out using the primers listed in Table 1.

### Quantitative Real-time PCR (qRT-PCR)

Total RNAs prepared from RAW 264.7 cells using Accuzol (Bioneer Corporation) were reverse transcribed into cDNA and then qPCR was performed with iTaq Universal SYBR Green Supermix (Bio-Rad, Hercules, CA). The following primer sets were used: DUSP5 mRNA (forward, 5′-GAGTGCTGAGATTCTGTCCAG-3′; reverse, 5′-AAGTCCAAGGTCACCGAGGAAC-3′); GAPDH mRNA (forward, 5′-GCTCTCTGCTCCTCCTGTTC-3′; reverse, 5′-ACGACCAAATCCGTTGACTC-3′). The data obtained from qRT-PCR were quantified using the comparative threshold (2^−[*ΔΔ*Ct]^) method (Ct represents cycle threshold). Relative mRNA expression levels were normalized with GAPDH. The Ct value for each primer pairs was obtained from samples and averaged. Delta Ct value represented the calculated difference between the average Ct for the DUSP5 and the average Ct for GAPDH as the control for total starting RNA quantity. The delta-delta Ct method of calculation was the used to assess fold-change in gene expression relative to GAPDH gene.

### Luciferase assay

HEK 293 cells seeded into a 100-mm dish were transfected with 4.5 μg pNF-κB-Luc or pAP-1-Luc cis-reporter plasmids (Agilent Technologies, Inc., Santa Clara, CA, USA) for 6 h at 37 °C. The gWIZ-green fluorescent protein (GFP) plasmid was used as an internal control for normalization. Transfected cells were split into 12-well plates and further transfected with or without FLAG-tagged DUSP5 expressing plasmids using OmicsFect. After 32 h of transfection, cells were treated PMA for additional 16 h. Cells were lysed with Cell Culture Lysis Reagent (Promega, Madison, WI) and then extracted samples were analyzed with Luciferase Assay Reagent (Promega). Luminescence from the product was measured with a multiwall plate reader (Synergy H1: Luminometer filter). Relative fold induction of luciferase activity was determined and normalized to GFP. All luciferase assays were repeated at last three times.

### Knockdown of DUSP5

For RNA interference of DUSP5, RAW 264.7 cells grown at 40% confluences were transfected with 50 nM of scrambled negative control siRNA or 50–100 nM of DUSP5 siRNAs [#1: 5′-CUC ACA AGA GAA GAU CGA A(dTdT), #2: 5′-AGA CCU UCU ACU CAC AGU A(dTdT)] (Bioneer Corporation) using Neon Transfection System (Invitrogen). The negative control siRNA used was purchased from Bioneer. After 48 h of transfection, cell lysates were prepared and subjected to immunoblotting analysis with an anti-DUSP5 antibody.

### Statistical analysis

All the figures described here are representative of at least three experimental repeats. For statistical analysis, differences between experimental conditions were assessed by the Student’s t-test. p < 0.05 was considered statistically significant. At all instances, the means of data from three independent experiments were analyzed.

## Electronic supplementary material

Supplementary Figures
