# Supplementary material for: Dual-specificity phosphatase 5 acts as an anti-inflammatory regulator by inhibiting the ERK and NF-κB signaling pathways
Source: Sci Rep. 2017 Dec 11;7:17348. doi: 10.1038/s41598-017-17591-9 (PMC5725455; doi:10.1038/s41598-017-17591-9)
Supplement: Supplementary file 1 — Supplementary Figures [file 41598_2017_17591_MOESM1_ESM.pdf]

Dual-specificity phosphatase 5 acts as an anti-inflammatory regulator by inhibiting the ERK and NF-κB signaling pathways

Huiyun Seo, Young-Chang Cho, Anna Ju, Sewoong Lee, Byoung Chul Park, Sung Goo Park, Jeong-Hoon Kim, Kwonseop Kim, and Sayeon Cho

SUPPLEMENTARY INFORMATION

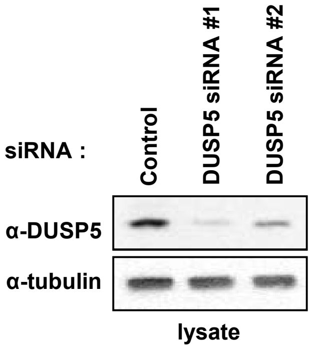

Supplementary Figure 1. Knockdown of DUSP5. After transfection with control or *DUSP5* siRNAs (#1 and #2), *DUSP5* knockdown was confirmed by immunoblotting using anti-DUSP5 and anti-tubulin antibodies.

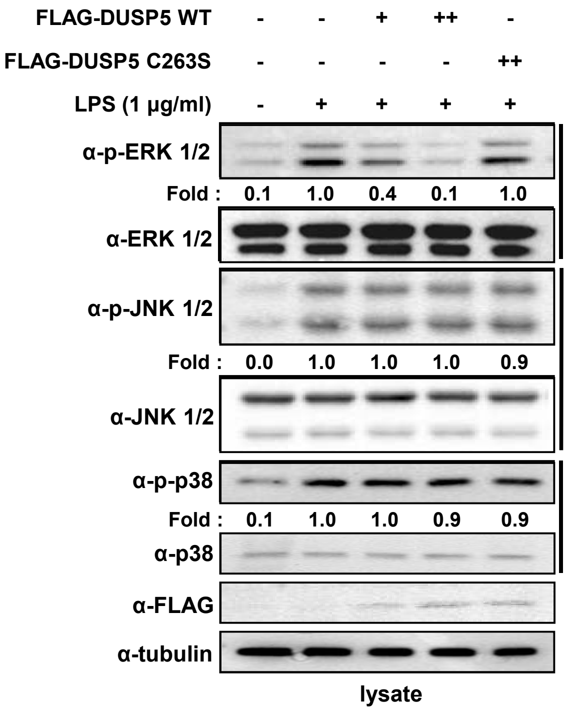

Supplementary Figure 2. Regulation of ERK phosphorylation by DUSP5. After RAW 264.7 cells transfected with DUSP5-WT or -C263S expression plasmids were stimulated with LPS (1 μg/ml) for 30 min, immunoblotting was performed for total and phosphorylated proteins as indicated. Relative phosphorylation levels of MAPKs were normalized to the expression levels of the corresponding total MAPKs and are presented as fold increases. Similar results were obtained in three independent experiments.

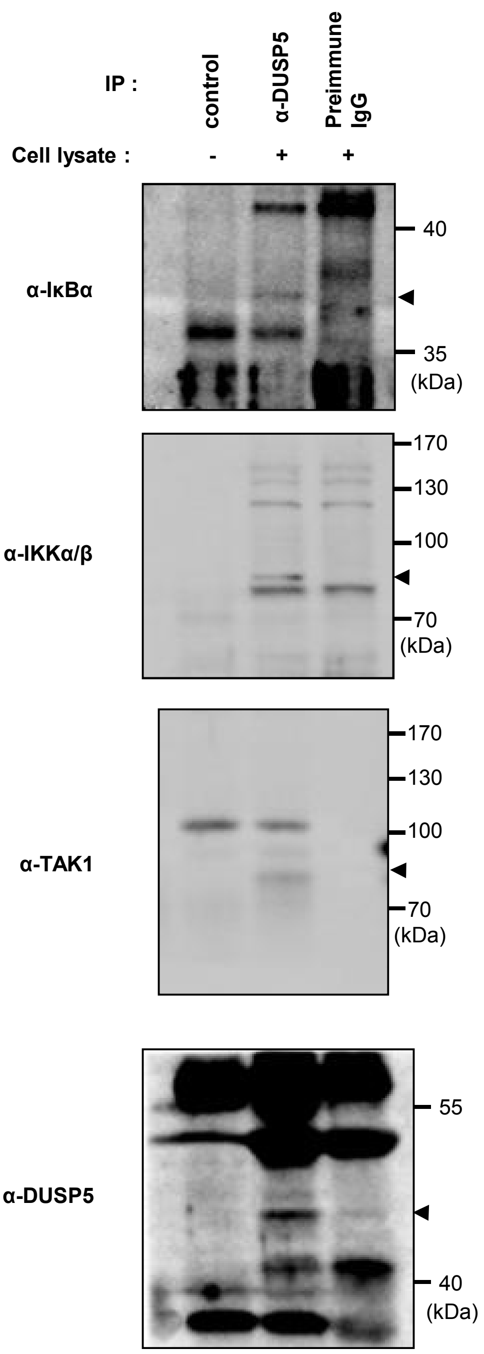

Supplementary Figure 3. The original immunoblotting data (full blot) of immunoprecipitation assays used in Fig. 5a. Molecular weight markers are labeled on the right. Specific protein bands are indicated by arrow heads.

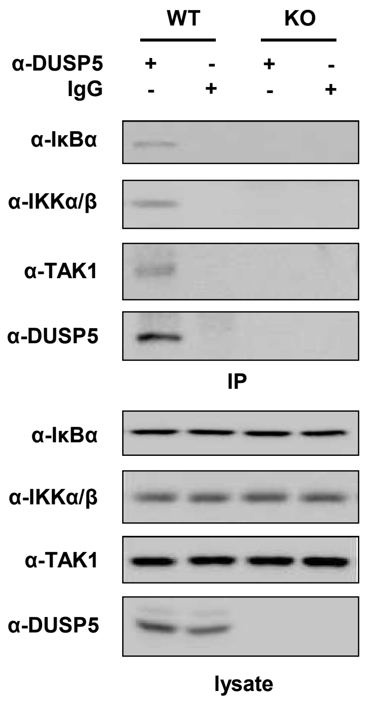

Supplementary Figure 4. Interaction between DUSP5 and NF-κB signaling axis in *DUSP5* WT and KO MEFs. Cell lysates from *DUSP5* WT and KO MEFs were immunoprecipitated with goat preimmune IgG or anti-DUSP5 antibody, then incubated with protein A/G beads. Bound proteins were identified with anti-IκBα, anti-IKKα/β, and anti-TAK1 antibodies. Similar results were obtained in three independent experiments.

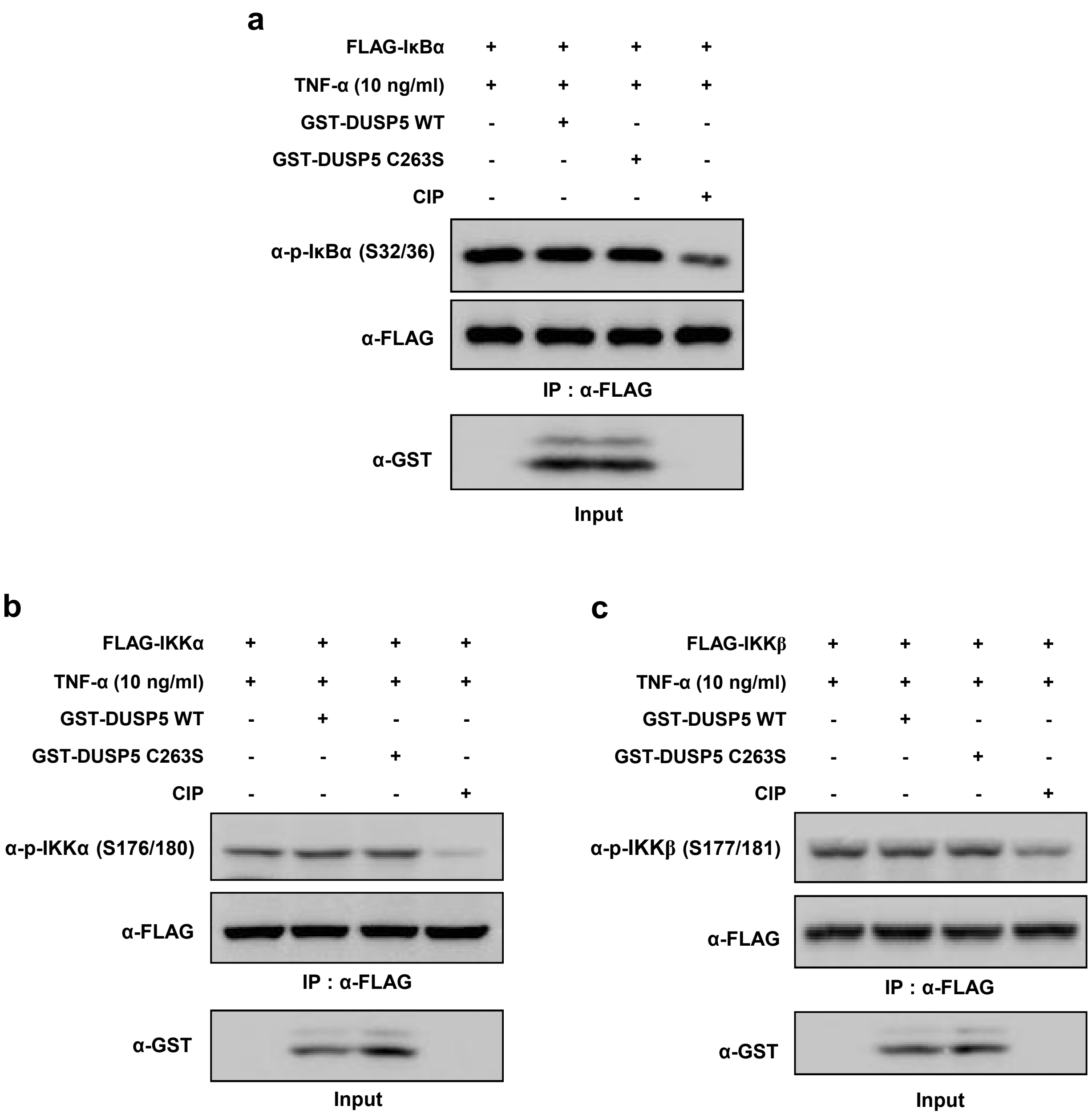

Supplementary Figure 5. Effect of DUSP5 WT and C263S mutant on the dephosphorylation of p-IκBα, p-IKKα, and p-IKKβ *in vitro*. After HEK 293 cells expressing FLAG-IκBα (a), -IKKα (b), or –IKKβ (c) were stimulated with 10 ng/ml TNF-α for 10 min, total cell lysates were immunoprecipitated with anti-FLAG M2 agarose for 3 h and incubated with recombinant DUSP5 proteins for 1 h, followed by immunoblotting analysis. The levels of p-IκBα (a), p-IKKα (b), or p-IKKβ (c) were determined using appropriate antibodies. Data are representative of three independent experiments. Calf-intestinal alkaline phosphatase (CIP) was used to confirm dephosphorylation of phosphorylated proteins.
